# Augmented reality and radiology: visual enhancement or monopolized mirage

**DOI:** 10.1093/bjro/tzae021

**Published:** 2024-08-20

**Authors:** Matthew Christie

**Affiliations:** Queen Alexandra Hospital, Portsmouth Hospitals University NHS Trust, Cosham, Portsmouth PO6 3LY, United Kingdom

**Keywords:** augmented reality, virtual reality, mixed reality, enhanced reality, artificial intelligence, fusion imaging

## Abstract

Augmented reality (AR) exists on a spectrum, a mixed reality hybrid of virtual projections onto real surroundings. Superimposing conventional medical imaging onto the living patient offers vast potential for radiology, potentially revolutionising practice. The digital technology and user-interfaces that allow us to appreciate this enhanced environment however are complex, expensive, and development mainly limited to major commercial technology (Tech) firms. Hence, it is the activity of these consumer-based businesses that will inevitably dictate the available technology and therefore clinical application of AR. The release of mixed reality head-mounted displays in 2024, must therefore prompt a review of the current status of AR research in radiology, the need for further study and a discussion of the complicated relationship between consumer technology, clinical utility, and the risks of monopolisation.

## Introduction

A narrative review was conducted of the literature to explore existing uses and emerging applications of augmented reality (AR) in radiology.

In recent years, driven by miniaturization of technology and development, principally in the gaming industry, evolution in clinical applications of AR has been rapid, with projected expenditures of $5.1bn by mid-decade.^1^

Early technological constraints and resulting lack of anatomical realism made limited virtual reality (VR) simulations, whereby visual appreciation of the real-life objects or “reality” is completely replaced by a virtual construct, more viable, and better-suited to rudimentary procedural training or patient-as-user formats. Facilitating informed consent and distraction/relaxation techniques saw their introduction to radiology workflow and image acquisition, reducing MRI-claustrophobia/anxiety and decreasing paediatric sedation/anaesthetic requirements.^2^

**Figure 1.**
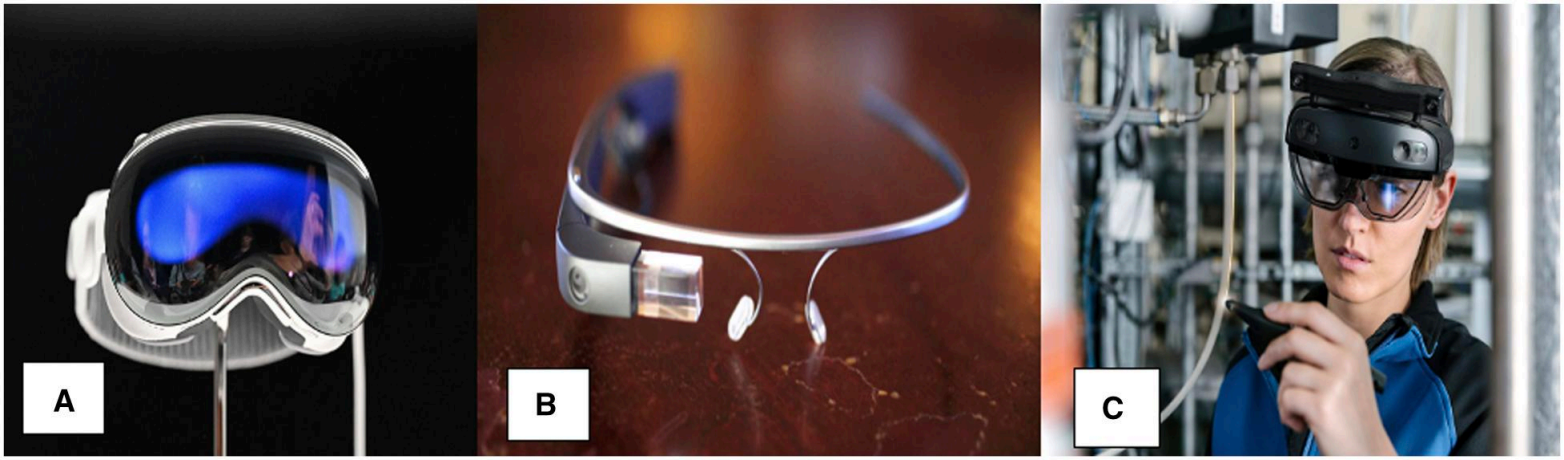
Augmented reality interfaces: head-mounted displays. (A) Apple Vision Pro; License Details; Creator: Josh Edelson | Credit: AFP via Getty Images. (B) Google Glass; License Details; Creator: San Francisco Chronicle/Hearst Newspaper| Credit: San Francisco Chronicle via Getty Images | ONLINE_YES. (C) Microsoft Hololens 2; License Details; Creator: Simon Toplak | Credit: simontoplak.com | Copywright: © Simon Toplak.

Exponential improvements in computer-graphic power and user-interface designs have made AR’s more complex merger of virtual projections onto reality and clinician-as-user formats viable, appreciated through technologies such as head-mounted displays (HMDs) (Figure 1).^3^

Conventional radiological imaging can be synchronized and projected onto the living patient. AR offers enormous opportunities within radiology, enhancing our appreciation and utilization of medical imaging.

## The current status of AR within radiology

### Interventional

Interventional radiology (IR), endovascular or percutaneous, involves insertion of instrumentation from a puncture site, accurately directed to a target, under the guidance of conventional imaging modalities while minimizing radiation exposure.

**Figure 2.**
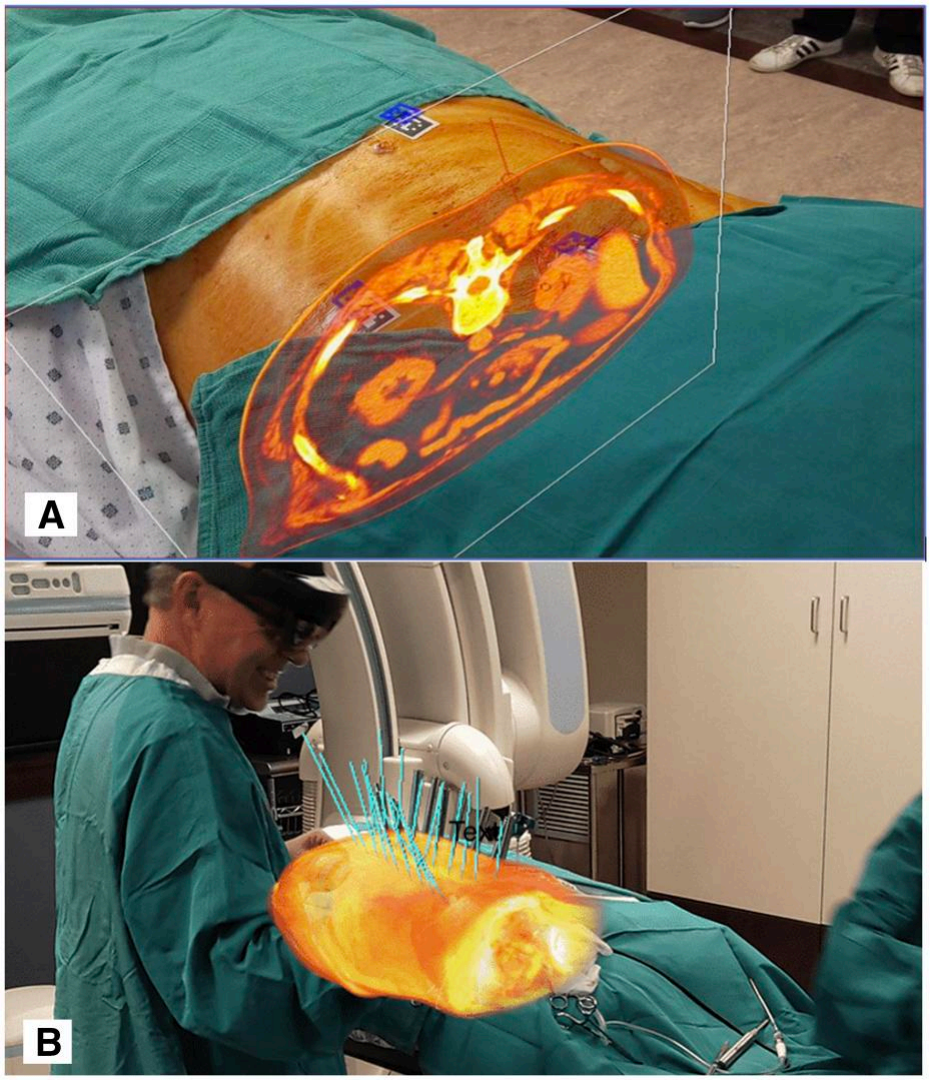
(A and B) Use of head-mounted displays to display conventional cross-sectional imaging modalities onto a live patient to facilitate spinal surgery (reproduced with permission from Novorad).

AR-based navigation systems superimpose virtual 3D anatomical projections generated from conventional pre-procedural cross-sectional imaging onto the patient (Figure 2). Software produces a virtual device trajectory overlaid onto visual surface anatomy, facilitating accurate navigation to target. Theoretically, this removes the need for conventional, intraprocedural image-guidance, seeing application to spinal injections, arthrograms even vertebroplasty.^3^^,^^4^

Within a rapidly evolving field, interventional oncology procedures such as biopsy and tumour ablation can be performed with greater accuracy and decreased exposure to ionizing radiation (Table 1).^5^

**Table 1.** Descriptive statistical analysis of the 3 major endpoints for percutaneous lung biopsy (PLB) standard CT guidance versus SIRIO (Sistema robotizzato assistito per il puntamento intraoperatorio)—an augmented reality (AR)-assisted CT navigation technique.^5^

Variable	SIRIO (AR-assisted) CT-guided PLB		Standard CT-guided PLB		*P*-value	ΔX% (reduction with SIRIO)
	Mean	SD	Mean	SD		
Time (min)	26.4	9.7	37.3	11	<.001	29%
No. of scans	5.9	2.4	10.3	4.1	<.001	43%
Dose (mSv)	6.1	2.9	12.2	5.4	<.001	50%

In early clinical trials, AR-assisted image-guided biopsies consistently outperformed their conventional image-guided equivalent in obtaining tissue samples adequate for histopathological diagnosis of lung nodule biopsies, proving especially beneficial with smaller targets and longer procedural distance of travel (Table 2).^5^

**Table 2.** Accuracy of AR (SIRIO)-assisted CT-guided PLB versus standard CT-guidance depending on size of lesion.^5^

Lesion size	SIRIO (AR-assisted) CT guided PLB	Standard CT-guided PLB
>20 mm	93.7%	92.3%
≤20 mm	96.8%	91.4%

Abbreviations: AR = augmented reality; PLB = percutaneous lung biopsy; SIRIO = Sistema robotizzato assistito per il puntamento intraoperatorio.

Superior sample adequacy associated with AR-assisted navigation is replicated in other image-guided biopsies both for other organs (CT-Renal) and imaging modalities (MRI-bone).^6^

**Figure 3.**
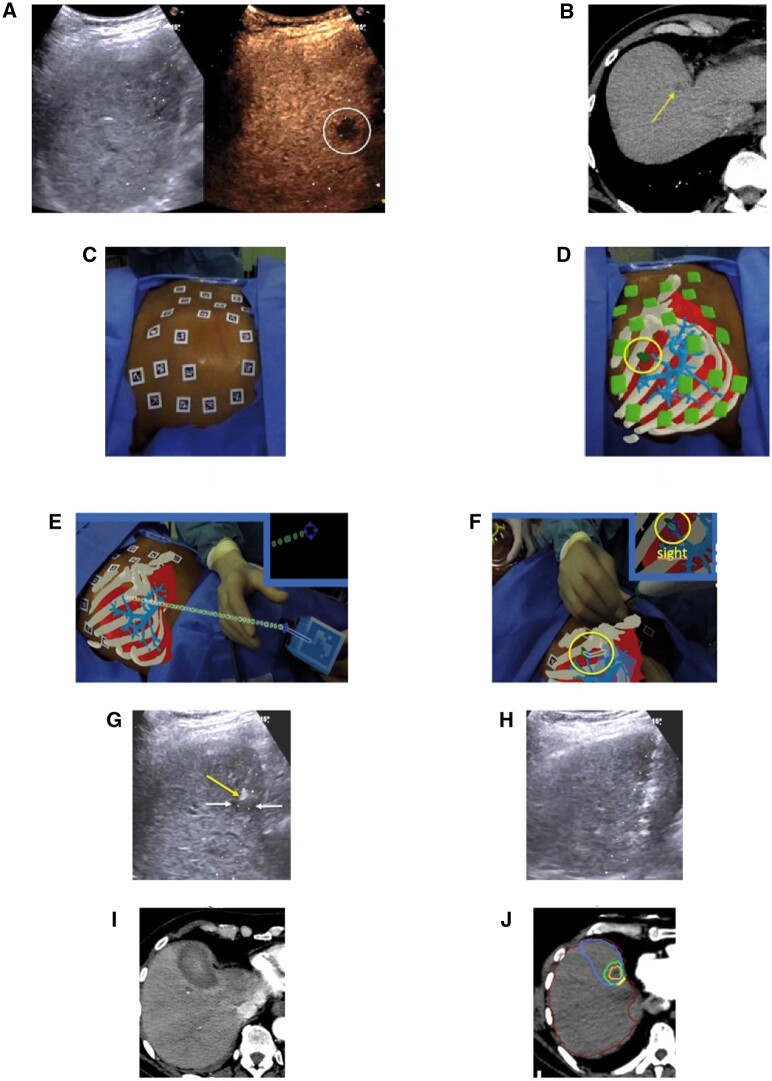
Thermal ablation guided by augmented reality (AR); a 1.5-cm metastasis in segment VIII of the liver from a pancreatic carcinoma primary, difficult to visualize on B-mode ultrasound (US) (A) [left] but clearly seen by contrast-enhanced ultrasound (A) [right] as well as on contrast-enhanced CT performed pre-ablation(arrow) (B). Radiopaque AruCo fiduciary markers applied to the patient’s skin overlying the zone of interest (C). The AR view generated by the head-mounted display (HMD) and visible to the clinician: ribs (white), liver (red), major hepatic vessels (light blue), and the target lesion (green, surrounded by a yellow circle) (D). The HMD view allows the clinician to appreciate the virtual needle (blue) and the optimal trajectory line, connecting the needle tip to the target lesion centre (green) (E). Utilizing the optimal trajectory line generated facilitates successful lesion (tumour) targeting with AR guidance alone (F). The US shows a 5.4 mm gap from the coaxial needle tip and the centre of the target (G). A microwave antenna is then introduced into the coaxial needle (H). A significant volume of ablation, entirely surrounding the targeted metastasis can be visualized on the post-ablation CT. (I) Using confirmatory software (Ablation-fitTM) shows the technical success of the ablation. Margins of target metastasis (orange), 5-mm ablation margin (green), and necrotic volume margins (blue) on CT. Using this AR-guided technique, complete tumour ablation was attained with just 5.4% of the safety margin outside of the necrotic volume (J).^7^ (A-J) Reproduced from Solbiati et al.^7^

The first in vivo study of hepatic tumour ablation solely guided by AR, involved 8 patients with 15 tumours (9 hepatocellular carcinomas and 6 metastases of mixed primaries). Contrast-enhanced CTs were used to create 3D virtual reconstructions, overlayed and synched to the patient with AruCo code markers and the optimal probe trajectory to the target lesion was automatically plotted. Probe position was confirmed by conventional CT-imaging prior to ablation. The study found rapid AR system setup and procedural targeting times in addition to high targeting accuracy and technical success in all lesions (ie, complete tumour ablation and 13/15 with >90% 5-mm peri-ablation margin) (Figure 3).^7^

### Diagnostic

Future diagnostic applications of AR are less clear and seemingly VR rather than AR will predominate. In breast imaging, AR variants have shown promise enhancing the detection of microcalcifications and spiculations, enabling virtual transparency of overlying anatomy with joystick fly-throughs and improving early diagnosis of breast cancer-related lymphoedema.^8^^,^^9^

AR-enabled, dynamic, multi-modality image fusion may also enable more comprehensive assessments, aiding diagnosis by combining optimal modalities to visualize specific anatomy or providing spatial awareness during ultrasound (US).

In contrast, diagnostic interest in VR often centres on “virtual reading rooms,” potentially overcoming physical space limitations within departments and optimizing conditions for image interpretation, including 360° appreciation of complex anatomy. VR headsets have been used to recreate traditional screen displays and ambient lighting conditions of reporting rooms that could be manipulated using hand gestures. This could increase efficient use of reporting space, benefiting trainees who cite this as an issue in expanding training capacity^10^ or ensuring standardized reporting environments, particularly as home reporting grows in popularity.

### Communication/collaboration

Radiology is a highly collaborative and patient-facing specialty. AR may facilitate inter-speciality assistance with multi-disciplinary team meetings or surgical planning and benefit patient communication by contextualizing otherwise complex imaging findings or procedures. AR has the potential to revolutionize remote support, be it consultant input for trainees (especially on-call), through to highly specialist input that would otherwise be geographically impossible, through annotation or manipulation of virtually projected anatomy for diagnostic or interventional guidance. Parallel advances in artificial intelligence might one day offer live interventional support in the form of annotation, analysis, differentials, and prognoses alongside AR projections.

### Educational

**Figure 4.**
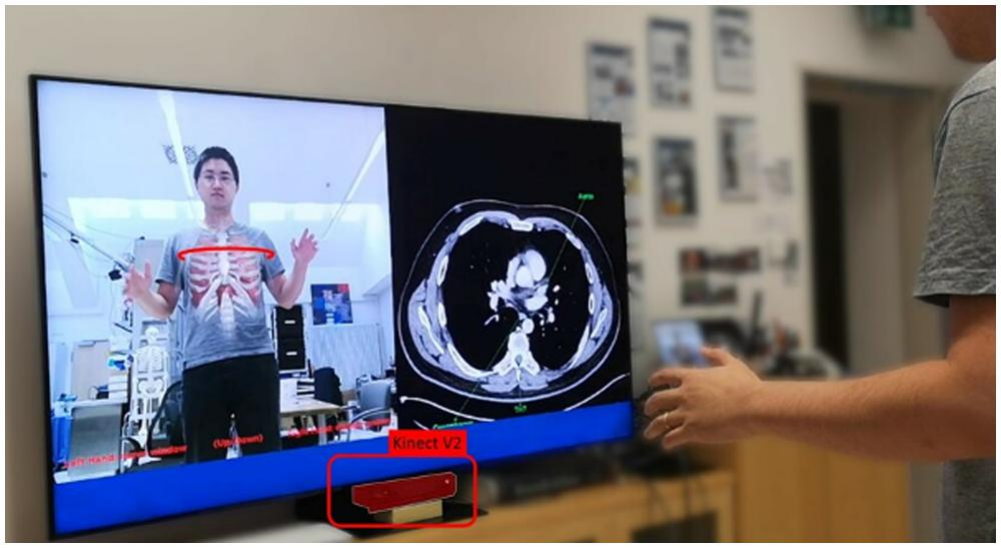
The so-called “Magic Mirror” shows the inner anatomy on the user’s “reflection” captured by an RGB-D camera. Alongside this projection, the mirror also shows transverse CT slices that can be selected by the user through hand gestures. Additionally, the pose of the user in front of the mirror distorts the virtual reflection of the user’s anatomy as it appears on the magic mirror.^11^

The potential applications of AR in medical education and radiology training are huge. As a supplement to conventional dissection, 880 medical students utilized an AR “Magic Mirror” displaying radiological cross-sectional anatomy onto the user’s reflection, the plane and slice level manipulated by hand gestures (Figure 4). Students reported improved appreciation of the 3D nature and courses of anatomical structures, especially deep, delicate, or complex anatomy.^12^ Similar findings have been replicated using donor-specific AR overlay of cadaveric imaging, with students reporting improved spatial awareness of anatomical structures and higher dissection grades.^13^

Potential educational benefits of AR extend to radiology trainees. AR may improve understanding of complex anatomy or pathology on conventional imaging by enabling simultaneous overlay/side-by-side viewing of 3D image reconstructions or illustrating 2D transection plane on a patient during US. AR may increase access to simulation-based training or supervision that is otherwise resource heavy or limited by opportunity.

Utilization of AR in IR training has shown higher levels of participation compared to textbooks and online resources because of trainees’ increased social, environmental, and personal immersion within the learning activity.^14^

### Current limitations and future research

Significant technological issues affect AR, curbing its radiological application. Accurate tracking and registration, allowing for the movement and gaze of the user while maintaining synchronized projection of images onto a live patient is complex, complicated further by patient movement, respiration, and organ deformation.^15^ Research into respiratory-gating, non-rigid end devices and fiduciary markers is ongoing.

AR-supplementation relies on spatial and temporal fidelity of projected anatomy, interval change due to evolving pathologies such as ischaemic progression and associated haemorrhagic risk cannot be underestimated. In certain situations, US might be able to provide live data as feedback to dynamically alter/update AR-projections. The user’s ability to identify situational change must be paralleled by the ability to interact with or manipulate the AR environment intuitively, reducing training demands and allowing rapid responses in emergencies.

Prolonged HMD use has also been associated with nausea attributed to vestibulo-ocular discrepancy and headsets can be uncomfortable.^16^ Technology remains expensive, with reporters already familiar with conventional imaging software, the need for a “virtual reading room” and whether it would increase reporting capacity is questionable.

Unfortunately, to date, most studies on AR-supplemented radiology have been small proofs-of-concept and we must now progress to higher-powered, higher-quality of evidence from large, multi-centre randomized controlled trials.

## The AR monopoly and radiology consumer

At present AR technologies are highly experimental and hardware is not designed with a radiological mandate. We are merely consumers of technology, whose primary audience and obligations are to the gaming industry. This makes the commercial release of consumer AR hardware highly relevant to research into the radiological applications of AR. However, it should also prompt discussion about the potential dangers of this consumer relationship with AR technology and the risks of monopolization by tech giants.

The extensive resources, financial and expertise, required for research and development of AR technology are prohibitive to all but a few companies and afford them massive influence over the direction of AR innovation and progress. Their priorities will not always align with radiology and differences in safety standards, margins of error and data security may pose issues.

However, consumer technology is cheaper due to economies of scale, prioritizing user-friendliness and ease of integration with pre-existing technologies. Moreover, utilizing AR in radiology could provide tech giants with a reliable source of revenue from AR, including long-term support with software updates and maintenance. This may compel tech companies to involve radiologists in research and development of AR. Through advocacy and collaboration, radiologists can ensure that clinical needs are better addressed.

## Conclusion

AR shows great potential in radiology, especially with intervention and education. Further work on tracking and registration is required. Research and development costs can be prohibitive and risk monopolization, but providing a reliable consumer base and increasing collaboration radiology can safeguard against this. Perhaps, the most complex interpretation is the distinction between the reality of better hardware and monopolization of that very same technology.
